# Next-Generation Adaptogens: Molecular Mechanisms, Bioactive Compounds and Human Health Relevance of Selected Botanicals and Fungi

**DOI:** 10.3390/ijms27198565

**Published:** 2026-09-25

**Authors:** Sebastian Such, Maria Czernicka

**Affiliations:** Department of Bioenergetics, Food Analysis and Microbiology, Faculty of Technology and Life Sciences, University of Rzeszów, Zelwerowicza 4 St., 35-601 Rzeszow, Poland; ss141887@stud.ur.edu.pl

**Keywords:** adaptogens, allostatic load, phytochemicals, AMPK, telomerase, endocannabinoid system, neuroplasticity, phytoecdysteroids, 11β-HSD2

## Abstract

Adaptogens are natural substances proposed to enhance non-specific resistance to stress and support physiological homeostasis. This review critically evaluates the phytochemistry, molecular mechanisms, human-health relevance, and translational evidence for seven proposed next-generation adaptogens: *Lepidium meyenii*, *Astragalus membranaceus*, *Cordyceps militaris*, *Rhaponticum carthamoides*, *Gynostemma pentaphyllum*, *Centella asiatica*, and *Glycyrrhiza glabra*. Evidence from mechanistic studies, clinical trials, systematic reviews, and meta-analyses was critically synthesised with emphasis on chemically characterised preparations, molecular target engagement, human exposure, and the distinction between preclinical and clinical evidence. The most consistently supported mechanisms include fatty acid amide hydrolase (FAAH) inhibition by maca macamides; telomerase- and senescence-related effects of *Astragalus* constituents; and AMP-activated protein kinase (AMPK)-centred metabolic regulation by cordycepin and gypenosides. Other relevant pathways include oestrogen receptor beta (ERβ)-related and phosphoinositide 3-kinase/protein kinase B (PI3K/Akt)-dependent anabolic signalling by phytoecdysteroids; brain-derived neurotrophic factor/tropomyosin receptor kinase B (BDNF/TrkB)-associated neuroplasticity and nuclear factor erythroid 2-related factor 2 (NRF2)-dependent redox regulation by *C. asiatica*; and 11β-hydroxysteroid dehydrogenase type 2 (11β-HSD2) inhibition by glycyrrhetinic acid. However, evidence strength differs markedly among species and outcomes, and many mechanisms remain predominantly preclinical. Clinical translation is further limited by extract variability, incomplete pharmacokinetic characterisation, uncertain bioavailability, and safety concerns. The next-generation adaptogen concept should therefore be regarded as a pathway-oriented framework whose validation requires standardised preparations, mechanism-aligned biomarkers, clinically relevant endpoints, and rigorous safety assessment.

## 1. Introduction

Human physiology is continuously challenged by interacting metabolic, infectious, psychosocial, environmental, and lifestyle-related stressors. Acute activation of the hypothalamic–pituitary–adrenal (HPA) axis and sympathoadrenal system mobilises energy substrates, modifies immune activity, and supports short-term adaptation. When these responses become recurrent, prolonged, or insufficiently terminated, however, the same mediators may contribute to cumulative physiological strain and progressive loss of regulatory flexibility [1,2]. The concept of allostasis describes the maintenance of stability through adaptive change, whereas allostatic load denotes the cumulative biological cost of repeated or sustained adaptation. The persistent disruption of stress-system regulation has been associated with cardiometabolic dysfunction, impaired glucose homeostasis, neurodegeneration, immune dysregulation, chronic fatigue, and depressive disorders [3,4,5]. These interrelated outcomes provide a biological rationale for interventions capable of supporting resilience without producing an excessive stimulation of already activated stress pathways.

Against this background, plant- and fungal-derived adaptogens have attracted renewed scientific interest as potential modulators of stress resilience and physiological homeostasis. The modern pharmacological definition requires an adaptogen to be relatively safe, to increase non-specific resistance to diverse stressors, and to exert a normalising rather than strictly unidirectional effect on physiological function [6]. The concept has nevertheless evolved beyond its original emphasis on general resistance. Contemporary interpretations increasingly frame adaptogenic activity as network-level regulation involving neuroendocrine, inflammatory, metabolic, and cellular stress-response pathways [6,7]. This expansion is scientifically attractive but also creates a risk of conceptual overextension, because chemically heterogeneous extracts are sometimes classified as adaptogens on the basis of historical use or broad bioactivity rather than reproducible pharmacological evidence. Accordingly, mechanistic plausibility, extract standardisation, exposure, safety, and clinically meaningful outcomes must be evaluated together rather than treated as interchangeable indicators of adaptogenic efficacy [8].

Classical adaptogens, particularly *Panax ginseng* C.A.Mey., *Eleutherococcus senticosus* (Rupr. & Maxim.) Maxim., and *Rhodiola rosea* L., are most commonly associated with modulation of the HPA axis, sympathoadrenal activity, stress-responsive proteins, redox balance, and inflammatory signalling [6,9]. These mechanisms remain central to the established adaptogen framework, but they represent only part of the molecular repertoire through which natural compounds may influence the consequences of chronic stress [7,8]. A broader, pathway-oriented perspective has therefore emerged in which candidate next-generation adaptogens are differentiated by more specific biochemical entry points, including endocannabinoid turnover and telomere maintenance [10,11], AMP-activated protein kinase (AMPK) signalling, mitochondrial biogenesis, glucose and lipid metabolism [12,13], neurotrophin-dependent plasticity [14], non-androgenic myogenic signalling [15], and peripheral glucocorticoid metabolism [16,17]. Several of these pathways also intersect with mechanisms relevant to cellular ageing and the maintenance of functional resilience, thereby linking chronic stress adaptation with age-related cellular dysfunction and healthy longevity. The term next-generation adaptogen is used here as an analytical framework rather than as a formally recognised pharmacological category, because the strength of supporting evidence varies markedly among compounds, preparations, mechanisms, and health outcomes [7,8]. Within this framework, candidate adaptogens were considered on the basis of relative safety, capacity to support non-specific resistance and physiological normalisation, evidence for a defined molecular mechanism relevant to stress adaptation or homeostatic regulation, and sufficient phytochemical and translational characterisation to permit critical evaluation of exposure, efficacy, and safety. These criteria were used to identify mechanistically informative candidates rather than to imply equivalent evidential strength or formal adaptogen status.

The seven species examined in this review were selected to represent complementary molecular strategies extending beyond the conventional HPA centred model and to provide sufficient mechanistic, phytochemical, human, and safety evidence for critical translational comparison. The selection was therefore intended to capture mechanistic diversity rather than to provide an exhaustive catalogue or ranking of botanicals and fungi with proposed adaptogenic properties. In *Lepidium meyenii* Walp., macamides have been linked to the inhibition of fatty acid amide hydrolase and modulation of endogenous cannabinoid tone, although direct pharmacodynamic confirmation in humans remains limited [10,18,19,20]. *Astragalus membranaceus* (Fisch.) Bge. contains astragalosides, cycloastragenol, polysaccharides, and flavonoids associated with immune regulation, telomere biology, and cellular senescence; however, the clinical relevance and long-term safety of telomerase activation require cautious interpretation [21,22,23]. *Cordyceps militaris* (L.) Link represents a metabolically oriented profile in which cordycepin and fungal polysaccharides have been connected with AMPK signalling, mitochondrial function, inflammatory regulation, and physical performance [24,25,26].

Complementary mechanistic domains are represented by the remaining four species. Phytoecdysteroids from *Rhaponticum carthamoides* (Willd.) Iljin, particularly 20-hydroxyecdysone, have been associated with ERβ-dependent PI3K/Akt/mTOR signalling and skeletal-muscle protein synthesis without classical androgen-receptor activation [15,27,28]. Gypenosides from *Gynostemma pentaphyllum* (Thunb.) Makino are principally considered in relation to the LKB1/AMPK-dependent regulation of glucose uptake, lipogenesis, and hepatic lipid handling [29,30,31]. Triterpenes from *Centella asiatica* (L.) Urb., including asiaticoside and madecassoside, have been linked to BDNF/TrkB/CREB signalling, antioxidant defence, neuroplasticity, and cognitive outcomes [14,32,33,34]. Glycyrrhizin and its metabolite glycyrrhetinic acid from *Glycyrrhiza glabra* L. influence peripheral glucocorticoid availability through the inhibition of 11β-hydroxysteroid dehydrogenase type 2. This mechanism may prolong local cortisol action but simultaneously illustrates the narrow boundary between a potentially adaptive effect and clinically relevant toxicity, including sodium retention, potassium loss, hypertension, and herb–drug interactions [17,35,36]. The principal bioactive compounds and proposed molecular targets through which these species may influence allostatic regulation are summarised in Figure 1.

Each species is subsequently evaluated according to its phytochemical composition, experimentally supported molecular mechanisms, reported health-related effects, safety considerations, and the strength of the available human evidence. Particular attention is given to the distinction between biochemical activity demonstrated in isolated systems, efficacy observed in animal models, and clinically meaningful outcomes in humans. The review also considers chemical standardisation, bioavailability, pharmacokinetic uncertainty, herb–drug interactions, and the limitations of extrapolating from isolated compounds to complex botanical or fungal preparations. Rather than cataloguing beneficial effects, the analysis asks whether a defined preparation can be linked convincingly to a molecular target, a measurable biomarker, and a clinically relevant endpoint. In this way, the review evaluates whether the proposed next-generation adaptogens constitute a substantiated extension of adaptogenic pharmacology and nutraceutical science or remain mechanistically attractive candidates whose translational relevance is still incomplete.

## 2. Literature Search and Review Approach

The literature was evaluated according to the chemical definition of the intervention, level of experimental evidence, molecular plausibility, pharmacokinetic relevance, human translation, clinical relevance, and safety. Throughout the review, preclinical findings were distinguished from human mechanistic and clinical evidence, and mechanistic plausibility was not considered equivalent to demonstrated clinical efficacy. Literature searches were conducted using PubMed, Scopus, Web of Science Core Collection, and Google Scholar. The search covered publications available from database inception through the final update in June 2026. Only full text publications in English were considered.

Search terms were used individually and in combinations and included “adaptogen”, “adaptogenic”, “stress response”, “stress resilience”, “allostasis”, “allostatic load”, and the scientific and common names of the seven selected species. Species-specific searches were supplemented with terms referring to their principal bioactive constituents and relevant molecular mechanisms, including “macamides”, “FAAH”, “astragaloside IV”, “cycloastragenol”, “telomerase”, “cordycepin”, “AMPK”, “20 hydroxyecdysone”, “ecdysterone”, “PI3K/Akt/mTOR”, “gypenosides”, “damulin”, “asiaticoside”, “madecassoside”, “BDNF”, “NRF2”, “glycyrrhizin”, “glycyrrhetinic acid”, and “11β HSD2”. Additional searches combined these terms with “clinical trial”, “human”, “pharmacokinetics”, “bioavailability”, “metabolism”, “safety”, and “adverse effects”.

Priority was given to original experimental studies, controlled human studies, randomised clinical trials, systematic reviews, meta-analyses, and recent comprehensive reviews relevant to the phytochemical composition, molecular mechanisms, pharmacokinetics, human exposure, clinical effects, and safety of the selected species. Earlier studies were retained when they provided primary mechanistic evidence that remained relevant to the interpretation of the proposed molecular pathways. Reference lists of key primary studies and reviews were also examined to identify additional relevant publications.

Studies were included when the investigated botanical or fungal material, extract, fraction, isolated compound, or metabolite could be identified with sufficient clarity and when the reported findings contributed directly to the mechanistic, translational, pharmacokinetic, or safety questions addressed in this review. Evidence derived from whole botanical or fungal preparations, extracts, fractions, isolated compounds, and metabolites was evaluated separately and was not considered interchangeable. Particular attention was given to whether the investigated preparation was chemically characterised and whether the proposed molecular mechanism was supported by direct target engagement, pathway-related evidence, or downstream biological responses.

Publications were excluded when the identity of the investigated material was insufficiently described, when mechanistic claims could not be related clearly to the experimental model or intervention, when the paper did not provide information relevant to the defined scope of the review, or when only an abstract without sufficient methodological and experimental detail was available. Studies based exclusively on disease specific outcomes were retained only when they provided mechanistic or translational information relevant to stress response, physiological homeostasis, cellular resilience, or the molecular pathways considered in this review.

Because this was a narrative critical review, study selection was not intended to provide a quantitatively exhaustive synthesis of all available evidence. Instead, the literature was evaluated according to chemical definition of the intervention, level of experimental evidence, molecular plausibility, pharmacokinetic relevance, human translation, clinical relevance, and safety. Throughout the review, preclinical findings were distinguished from human mechanistic and clinical evidence, and mechanistic plausibility was not considered equivalent to demonstrated clinical efficacy.

## 3. *Lepidium meyenii* Walp. (Maca)

### 3.1. Phytochemical Composition

*Lepidium meyenii,* commonly known as maca or Peruvian ginseng, is a member of the Brassicaceae family native to the central Peruvian Andes, where it is traditionally cultivated at high altitudes, typically around 3500–4500 m above sea level [18,37]. The hypocotyl constitutes the principal edible and commercially utilised part of the plant and contains a chemically diverse matrix of primary and secondary metabolites. Early compositional studies identified substantial amounts of carbohydrates, proteins, lipids, fibre, minerals, fatty acids, and sterols, whereas subsequent phytochemical investigations revealed glucosinolates, alkaloids, macamides, macaenes, and other specialised metabolites [37,38,39].

Among the most characteristic constituents of maca are the macamides, which is a group of non-polar long-chain fatty acid *N*-benzylamides that have been regarded as distinctive chemical markers of *L. meyenii* [40,41]. Identified representatives include N-benzyl-(9Z)-octadecenamide, N-(3-methoxybenzyl)-(9Z)-octadecenamide, and N-benzyl-(9Z,12Z)-octadecadienamide, although the number and relative abundance of individual macamides vary considerably among samples. Importantly, macamide accumulation is strongly influenced by post-harvest processing. Evidence indicates that these compounds are scarce or absent in fresh intact hypocotyls and accumulate during drying, concomitantly with glucosinolate and lipid hydrolysis and the formation of benzylamine and free fatty-acid precursors [41,42]. Phytochemical profiles are additionally influenced by colour phenotype, geographical origin, cultivation conditions, and processing, although the contribution of individual factors is not always consistent across analytical studies [43,44].

### 3.2. Effects on Human Health

A systematic review of randomised clinical trials found limited evidence that maca may improve sexual function or sexual desire; however, only four randomised controlled trials (RCTs) met the inclusion criteria, and the small sample sizes and methodological limitations precluded firm conclusions [45]. Gonzales [18] similarly summarised clinical and experimental evidence suggesting potential effects of maca on sexual function while emphasising the broader and heterogeneous pharmacological evidence base.

Neuroprotective activity has been demonstrated predominantly in preclinical models. Pino-Figueroa et al. [46] reported a concentration-dependent neuroprotection of neurons exposed to oxidative stress in vitro and dose-dependent effects of maca extract in a rat model of cerebral ischaemia. More recently, Yu et al. [47] showed that a defined macamide attenuated corticosterone-induced hippocampal impairments in rats through anti-inflammatory, neurotrophic, and synaptic-protective mechanisms. Quispe-Díaz et al. [48] further demonstrated that a chemically characterised black maca extract reduced brain lipid peroxidation and improved spatial learning in ovariectomised rats, providing additional experimental evidence of neuroprotective activity. Nevertheless, evidence for cognitive and neuroprotective efficacy in humans remains limited, and mechanistic attribution should remain cautious. These observations provide a rationale for examining FAAH-dependent endocannabinoid signalling as a candidate molecular mechanism of maca, as discussed below.

### 3.3. Molecular Mechanisms

The best-supported molecular action of macamides is the inhibition of fatty acid amide hydrolase (FAAH, EC 3.5.1.99), which is a principal enzyme responsible for terminating anandamide (AEA) signalling [10,19]. By inhibiting FAAH, macamides may reduce AEA degradation and thereby prolong endogenous endocannabinoid signalling, although direct pharmacodynamic confirmation in humans is lacking. The molecular mechanism of maca provides the first example within a broader cross-species signalling framework, which is summarised in Figure 2 and subsequently revisited in the corresponding molecular-mechanism sections for the remaining adaptogens.

Almukadi et al. [19] provided direct biochemical evidence that *N*-(3-methoxybenzyl)-linoleamide acts as a time-dependent inhibitor of FAAH. Subsequent work confirmed FAAH inhibition by structurally related macamides and demonstrated that inhibitory potency depends on the structural features of the *N*-benzylamide moiety and fatty-acid chain [10]. These findings support FAAH inhibition as the most consistently demonstrated molecular target of macamides. Evidence for additional effects on endocannabinoid turnover is less conclusive. Rondón-Ortiz et al. [20] reported monoacylglycerol lipase (MAGL) inhibition and increased low-density lipoprotein receptor-related protein 1 (LRP1) expression in SH-SY5Y neuroblastoma cells following treatment with a maca-derived preparation; however, because these observations were obtained with a complex extract and reported in a conference abstract, they do not establish direct MAGL inhibition by individual macamides.

Taken together, these findings support FAAH inhibition as a plausible molecular entry point through which macamides may influence endocannabinoid signalling. However, direct evidence linking macamide-induced FAAH inhibition to subsequent cannabinoid receptor type 1 (CB1)- or cannabinoid receptor type 2 (CB2)-receptor activation in vivo remains limited. Preclinical studies nevertheless indicate that maca preparations and defined macamides can exert neuroprotective, anti-inflammatory, neurotrophic, and synaptic-protective effects [46,47,48]. Accordingly, the endocannabinoid pathway provides a mechanistically plausible framework for interpreting some of the reported neurological effects of maca, but the complete causal sequence from macamide exposure through endocannabinoid modulation to clinically meaningful outcomes has not yet been demonstrated in humans.

## 4. *Astragalus membranaceus* (Fisch.) Bge. (Huangqi)

### 4.1. Phytochemical Composition

*Astragalus membranaceus* (Fisch.) Bge., known as Huangqi in Traditional Chinese Medicine, belongs to the Fabaceae family. *Astragali radix* is the dried root of A. *membranaceus* or A. *membranaceus* var. *mongholicus* (Bge.) Hsiao and represents the principal medicinal raw material derived from these taxa [49,50]. Its phytochemical composition is highly complex and includes polysaccharides, cycloartane-type triterpenoid saponins, flavonoids and isoflavonoids, together with phenylpropanoids, alkaloids, amino acids, and other minor constituents [49,50,51]. Among the best-characterised bioactive fractions are *Astragalus* polysaccharides (APS), astragalosides, and isoflavonoids such as calycosin, formononetin, and their glycosides.

Astragaloside IV is one of the principal cycloartane-type saponins used as a chemical quality marker of *Astragali radix*. Structurally, it is a glycoside of cycloastragenol, whereas cycloastragenol represents an aglycone shared by several *Astragalus* cycloartane glycosides. The absolute content of astragaloside IV in root material is relatively low and varies with botanical source, cultivation conditions, processing, and analytical methodology; accordingly, pharmacopoeial specifications use astragaloside IV as a standardisation marker rather than as a quantitative representation of the entire saponin fraction [50]. *Astragalus* polysaccharides constitute a structurally heterogeneous group of water-soluble heteropolysaccharides whose molecular weight, monosaccharide composition, glycosidic-linkage pattern, and biological activity depend strongly on extraction and purification procedures [51]. This compositional heterogeneity complicates direct comparison among APS preparations and is particularly relevant when phytochemical characterisation is used to support subsequent mechanistic or clinical interpretation. Isoflavonoids, including calycosin, calycosin-7-O-glucoside, formononetin, and ononin, constitute another characteristic component of *Astragali radix* and contribute substantially to its chemically diverse small-molecule fraction [49,50].

### 4.2. Effects on Human Health

Clinical evidence for *A. membranaceus* is heterogeneous and is currently most developed in supportive oncology, whereas cardiovascular and anti-ageing applications are supported by a mixture of clinical and preclinical evidence. Recent systematic reviews and meta-analyses of randomised controlled trials suggest that *Astragalus*-based interventions may reduce cancer-related fatigue, improve quality of life, and favour selected immune indices in patients with cancer. Sheng et al. [52], analysing eight RCTs, reported improvements in cancer-related fatigue and quality of life, although the certainty of evidence was limited by small sample sizes, methodological weaknesses, and heterogeneity among studies. Similarly, a meta-analysis of 31 RCTs evaluating *Astragalus* polysaccharides reported improvements in several T-cell-related immune indices, including CD3+, CD4+, and the CD4+/CD8+ ratio, but it also highlighted a substantial variation in study quality and intervention protocols [53].

Cardiovascular effects of *Astragali radix* and its major constituents have been extensively investigated, although the mechanistic evidence remains predominantly preclinical. Su et al. [54] summarised anti-atherosclerotic, endothelial, cardioprotective, and anti-inflammatory effects associated with *Astragalus*-derived saponins and flavonoids, while more recent evidence reviews have emphasised the continued need for better-standardised clinical studies [50]. Human evidence related to telomere maintenance is more specific but remains limited. In a one-year randomised, double-blind, placebo-controlled trial involving 117 relatively healthy cytomegalovirus (CMV)-positive adults aged 53–87 years, Salvador et al. [55] reported a significant increase in median telomere length in the low-dose TA-65 group compared with telomere shortening in the placebo group. Notably, the higher TA-65 dose did not produce a statistically significant improvement, precluding a straightforward dose–response interpretation.

Borowicz and Jach [23] placed these findings within the broader context of cellular and systemic ageing while emphasising that the available human evidence remains insufficient to establish clinically meaningful anti-ageing effects of *A. membranaceus*. More specifically, Canale and Andreassi [56] reviewed telomere shortening as a component of vascular senescence and discussed astragaloside IV, cycloastragenol, and TA-65 as potential telomere-targeted interventions. However, the translational significance of telomerase activation remains uncertain because improvements in telomere-related biomarkers cannot yet be assumed to translate into reduced cardiovascular morbidity or extended healthspan.

### 4.3. Molecular Mechanisms

Cycloastragenol is best characterised as a telomerase-activating constituent of *Astragalus*. Telomerase is a ribonucleoprotein complex whose catalytic subunit, telomerase reverse transcriptase (TERT), contributes to maintenance of telomeric TTAGGG repeats. Because telomerase activity is low or absent in most differentiated somatic cells, the pharmacological modulation of this system has attracted considerable interest in the context of cellular senescence and ageing-related processes [11,22]. Mechanistic studies indicate that cycloastragenol can increase telomerase activity and TERT expression, although the upstream signalling mechanisms remain incompletely resolved and appear to involve several pathways, including Src/mitogen-activated protein kinase kinase (MEK)/extracellular signal-regulated kinase (ERK), Janus kinase (JAK)/signal transducer and activator of transcription (STAT), and CREB-dependent signalling [11]. Astragaloside IV exerts additional anti-inflammatory effects through the inhibition of nuclear factor kappa B (NF-κB) activation and adhesion-molecule expression [57,58]. Preclinical studies indicate that APS can influence Toll-like receptor 4 (TLR4)-dependent signalling, including myeloid differentiation primary response 88 (MyD88)/nuclear factor kappa B (NF-κB) and mitogen-activated protein kinase (MAPK) pathways, thereby modulating macrophage and other immune-cell responses and cytokine production [51]. Together, telomerase-related effects of cycloastragenol and the immunomodulatory and anti-inflammatory activities of other *Astragalus* constituents provide a mechanistic basis for the proposed positioning of *A. membranaceus* at the interface between adaptogenic and ageing-related pharmacology.

## 5. *Cordyceps militaris* (L.) Fr.

### 5.1. Phytochemical Composition

*Cordyceps militaris* (L.) Fr., commonly known as the scarlet caterpillar fungus, is an entomopathogenic ascomycete belonging to the Cordycipitaceae family. Unlike wild-harvested *Ophiocordyceps sinensis*, *C. militaris* can be cultivated under controlled conditions, including on cereal-based substrates, facilitating a more reproducible production of fruiting bodies and bioactive metabolites [25,59]. Cordycepin (3′-deoxyadenosine) is one of the principal bioactive nucleosides of *C. militaris* and differs from adenosine by the absence of the hydroxyl group at the 3′ position of the ribose. Its concentration varies markedly with fungal strain, cultivation substrate, and culture conditions. Other reported constituents include adenosine, mannitol (cordycepic acid), polysaccharides, ergosterol, carotenoids, proteins, amino acids, and other minor metabolites [25,60].

The polysaccharide fraction is structurally heterogeneous and includes intracellular and extracellular polysaccharides differing in monosaccharide composition, molecular weight, and glycosidic-linkage patterns. Although numerous preparations exhibit immunomodulatory, antioxidant, and other biological activities, relationships between polysaccharide structure and biological activity remain incompletely established [61].

### 5.2. Effects on Human Health

Human evidence for *C. militaris* remains limited with physical-performance studies providing suggestive but not yet definitive findings. In a randomised, double-blind, placebo-controlled trial, supplementation with 4 g/day of a mushroom blend containing *C. militaris* did not significantly improve maximal oxygen uptake (VO_2_max), ventilatory threshold, or time to exhaustion after one week. Among the smaller subset continuing supplementation for three weeks, improvements in VO_2_max and exercise tolerance were reported; however, the small sample size and use of a multi-mushroom preparation limit attribution specifically to *C. militaris* [62]. More recently, Farzan and Koushkie Jahromi [63] reported that a single 1 g dose of a standardised *C. militaris* extract increased resting oxygen uptake and improved post-exercise reaction time in recreationally active men but did not alter peak exercise VO_2_.

Preclinical evidence is broader. Lan et al. [13] demonstrated that cordycepin ameliorated non-alcoholic steatohepatitis in a murine model through AMPK activation and the improvement of hepatic lipid metabolism. Chai et al. [26] further reported anti-fatigue effects of cordycepin in mice associated with the regulation of energy metabolism and the TP53-induced glycolysis and apoptosis regulator (TIGAR)/sirtuin 1 (SIRT1)/peroxisome proliferator-activated receptor gamma coactivator 1-alpha (PGC-1α) pathway. These findings provide mechanistic support for metabolic and anti-fatigue effects but require further confirmation in adequately powered human trials.

### 5.3. Molecular Mechanisms

Cordycepin can activate AMP-activated protein kinase (AMPK) following cellular uptake and intracellular phosphorylation. Hawley et al. [64] demonstrated that cordycepin enters cells via adenosine transporters and is converted into mono-, di-, and triphosphate derivatives with AMPK activation correlating most closely with intracellular cordycepin monophosphate (CoMP). CoMP mimics the regulatory effects of AMP at the AMPK γ-subunit, thereby promoting AMPK activation. However, this intracellular mechanism must be distinguished from systemic exposure. Cordycepin undergoes rapid deamination to 3′-deoxyinosine, substantially limiting systemic exposure to the parent nucleoside [65,66]. Pharmacokinetic studies in rats have demonstrated the rapid depletion of cordycepin from circulation and showed that the inhibition of adenosine deaminase (ADA) with erythro-9-(2-hydroxy-3-nonyl)adenine (EHNA) suppresses its biotransformation and prolongs systemic exposure [66,67]. Rat pharmacokinetic studies further indicate short elimination half-lives and rapid blood clearance of cordycepin, reinforcing the importance of ADA-mediated metabolic inactivation when extrapolating preclinical activity to oral preparations [67]. Consequently, AMPK activation observed in cellular or animal models should not be assumed to occur to a comparable extent following the oral administration of *C. militaris* preparations in humans. AMPK activation is associated with phosphorylation of the catalytic α-subunit at Thr172 and shifts cellular metabolism towards ATP-generating pathways while suppressing energy-consuming anabolic processes (Figure 2) [12,64]. Downstream effects include the regulation of lipid metabolism and mitochondrial energy pathways. In experimental models, cordycepin-induced AMPK activation improves hepatic lipid handling [13], while cordycepin administration in a murine fatigue model increased SIRT1, PGC-1α, nuclear respiratory factor 1 (NRF1), and mitochondrial transcription factor A (TFAM) expression together with skeletal-muscle adenosine triphosphate (ATP) availability [26]. These findings support the interaction between energy-sensing and mitochondrial regulatory pathways.

Cordycepin also consistently suppresses inflammatory signalling and activates AMPK across a broad range of cellular and animal models [24]. However, the extent to which these molecular effects explain improvements in physical performance or other clinically relevant outcomes in humans remains unresolved.

## 6. *Rhaponticum carthamoides* (Willd.) Iljin (Maral Root)

### 6.1. Phytochemical Composition

*Rhaponticum carthamoides* (Willd.) Iljin (syn. *Leuzea carthamoides*), commonly known as maral root or Russian leuzea, is a perennial herb of the Asteraceae family native to mountainous regions of southern Siberia and Central Asia. The species is regarded as a particularly rich botanical source of phytoecdysteroids, which constitute a structurally diverse group of polyhydroxylated steroids related to arthropod ecdysteroids but occurring in plants as specialised secondary metabolites [7,28,68]. In addition to ecdysteroids, *R. carthamoides* contains flavonoids, phenolic acids, sesquiterpene lactones, triterpenoid glycosides, polyacetylenes, and volatile terpenoids with their relative abundance varying among roots, aerial tissues, and seeds [7,27,68].

The dominant and most extensively investigated phytoecdysteroid is 20-hydroxyecdysone (20E; also known as ecdysterone or β-ecdysone). Reported concentrations vary substantially according to plant organ, geographical origin, developmental stage, cultivation conditions, and analytical methodology; values reported for the roots are generally within approximately 0.04–1.7% of dry matter. Other ecdysteroids identified in *R. carthamoides* include polypodine B, inokosterone, ajugasterone C, makisterone derivatives, carthamosterone, and several less abundant structurally related compounds [7,68].

The non-ecdysteroid fraction further broadens the phytochemical profile of the species. Phenolic constituents include flavonoids and caffeoylquinic-acid derivatives, whereas sesquiterpene lactones and other terpenoid constituents contribute additional antioxidant, anti-inflammatory, and cytotoxic activities observed in experimental systems [27,68]. Comparative analyses of material cultivated in different geographical regions also demonstrate substantial variability in both secondary-metabolite composition and antioxidant activity, emphasising the importance of botanical origin and chemical standardisation when *R. carthamoides* preparations are used in pharmacological or clinical studies [7,27].

### 6.2. Effects on Human Health

The most frequently discussed health-related application of *R. carthamoides* concerns physical performance, skeletal-muscle adaptation, and recovery from exercise, which is largely because of the anabolic potential attributed to its phytoecdysteroid fraction. However, direct clinical evidence for chemically characterised *R. carthamoides* preparations remains limited, and evidence obtained with isolated 20-hydroxyecdysone should not automatically be extrapolated to the whole botanical extract [7,28,68]. Human studies of 20-hydroxyecdysone provide some evidence of anabolic activity, but the findings are not fully consistent. In a 10-week resistance-training intervention involving 46 young men, supplementation with a 20-hydroxyecdysone-containing preparation was associated with greater increases in muscle mass and one-repetition bench-press performance than the respective comparison conditions; routine markers of liver and kidney toxicity did not increase during the intervention [69]. In contrast, an earlier double-blind study in resistance-trained men found that 200 mg/day of 20-hydroxyecdysone for eight weeks did not significantly improve fat-free mass, strength, endurance, or circulating anabolic/catabolic hormone markers compared with placebo [70]. These contrasting findings indicate that the human ergogenic evidence remains limited and may depend on preparation, dose, exposure, training protocol, and study population.

Preclinical findings are more extensive. Roumanille et al. [71] demonstrated in a rat resistance-exercise model that acute *R. carthamoides* supplementation increased skeletal-muscle protein synthesis in selected muscles, whereas four weeks of supplementation increased mechanical power without consistently increasing muscle weight or fibre cross-sectional area. The study therefore supports an anabolic or performance-related signal but does not constitute evidence of clinical efficacy in humans. Moreover, the effects of combined *R. carthamoides* and *Rhodiola rosea* preparations cannot be attributed solely to either plant [71].

Beyond skeletal muscle, ecdysteroids from *R. carthamoides* have shown neuroprotective effects in experimental models. Wu et al. [72] reported reduced glutamate-induced hippocampal cell loss in rats together with the modulation of GRIN2B and increased PI3K/Akt/mTOR signalling. More recently, Nan et al. [73] demonstrated that *R. carthamoides* extract improved endothelial dysfunction and inflammatory responses in a mouse model of type 2 diabetes with effects involving sirtuin 6 (SIRT6) expression and Akt/endothelial nitric oxide synthase (eNOS) signalling. These findings broaden the potential biological relevance of the species but remain preclinical and should not be interpreted as evidence of neuroprotective or cardiovascular efficacy in humans.

The available safety evidence likewise warrants cautious interpretation. Experimental and short-term human studies suggest that 20-hydroxyecdysone does not act through the classical androgen receptor pathway and has not produced the characteristic androgenic profile associated with anabolic–androgenic steroids [15,28,69]. Nevertheless, the absence of an androgen-receptor-mediated mechanism does not establish long-term safety, and the clinical safety database for standardised *R. carthamoides* extracts remains substantially smaller than the preclinical literature.

### 6.3. Molecular Mechanisms

The anabolic activity of 20-hydroxyecdysone appears to involve oestrogen receptor beta (ERβ), although the available evidence does not justify describing ERβ as the exclusive molecular mediator. Parr et al. [15] demonstrated that 20-hydroxyecdysone-induced hypertrophy in differentiated C2C12 myotubes was inhibited by anti-oestrogenic treatment and by an ERβ-selective antagonist, whereas androgen-receptor antagonism did not suppress the response. Reporter assays in transfected cells further supported ER-mediated activity, and subsequent experimental and molecular-modelling studies reinforced ERβ as an important candidate mediator of 20-hydroxyecdysone-induced skeletal-muscle hypertrophy [15,28].

A complementary mechanism involves the rapid, non-genomic signalling upstream of Akt. Gorelick-Feldman et al. [74] showed that phytoecdysteroids increased protein synthesis in both C2C12 myotubes and primary human myotubes and that this effect was sensitive to PI3K inhibition. Subsequent experiments demonstrated that 20-hydroxyecdysone rapidly increased intracellular Ca^2+^, which was followed by sustained Akt activation and enhanced protein synthesis; these responses were attenuated by inhibitors of G-protein-coupled receptors, phospholipase C, and PI3K [74]. Together, these studies support the participation of membrane-associated signalling and the PI3K/Akt pathway rather than a simple classical nuclear-steroid-receptor mechanism.

Akt-dependent signalling can converge on the mTOR network, which regulates translational initiation, ribosomal activity, and muscle-protein synthesis. However, the available evidence does not support a single obligatory ERβ–PI3K/Akt–mTOR cascade as the mechanism underlying the anabolic effects of 20-hydroxyecdysone or *R. carthamoides* preparations. Gorelick-Feldman et al. [74] demonstrated the rapid Ca^2+^ mobilisation, Akt activation, and PI3K-sensitive stimulation of protein synthesis by isolated 20-hydroxyecdysone in cultured skeletal-muscle cells, establishing that this signalling route can operate under defined experimental conditions. By contrast, the increase in PI3K/Akt/mTOR signalling reported by Wu et al. [72] was observed in the rat hippocampus in association with neuroprotection and therefore cannot be regarded as direct evidence for the mechanism of skeletal-muscle anabolism. Importantly, Roumanille et al. [71] found that a *R. carthamoides* preparation increased acute skeletal-muscle protein synthesis in exercised rats without increasing the phosphorylation of Akt, mTOR, eukaryotic translation initiation factor 4E-binding protein 1 (4E-BP1), or ribosomal protein S6. This dissociation indicates that the measurable activation of the canonical Akt/mTOR pathway is not obligatory for the protein-synthetic response to a complex *R. carthamoides* preparation. Differences in the experimental model, tissue, timing of pathway assessment, and use of an isolated compound versus a multi-constituent botanical extract may therefore account for the apparently conflicting findings. Accordingly, the current evidence supports PI3K/Akt signalling as one experimentally demonstrated route through which 20-hydroxyecdysone may influence muscle protein synthesis, whereas downstream mTOR engagement should be regarded as context- and preparation-dependent rather than as a definitive universal mechanism [28,71,74].

Because *R. carthamoides* contains numerous additional ecdysteroids and phenolic constituents, effects observed with isolated 20-hydroxyecdysone should nevertheless be distinguished from those produced by chemically complex plant extracts [7,68].

## 7. *Gynostemma pentaphyllum* (Thunb.) Makino (Jiaogulan)

### 7.1. Phytochemical Composition

*Gynostemma pentaphyllum* (Thunb.) Makino, commonly known as Jiaogulan, is a climbing perennial herb of the Cucurbitaceae family native to East and Southeast Asia that is traditionally consumed as both a medicinal plant and herbal tea [75]. Its characteristic phytochemical feature is the abundance of dammarane-type triterpenoid saponins collectively known as gypenosides [31,75]. Although *Gynostemma* and *Panax* are taxonomically distant, several gypenosides are structurally identical or closely related to *Panax* ginsenosides, including compounds corresponding to ginsenosides Rb1, Rb3, Rc, Rd, F2, and Rg3 [75]. More than 200 individual gypenosides have been characterised from G. *pentaphyllum*, while an even larger number of dammarane-type saponins has been identified across the genus *Gynostemma* [31,75].

### 7.2. Effects on Human Health

Human evidence for *G. pentaphyllum* is most developed for glycaemic control, body composition, and selected aspects of physical performance, whereas evidence for direct hepatoprotective and neuroprotective effects remains predominantly preclinical [54,76]. In a randomised controlled study involving 24 drug-naïve patients with type 2 diabetes, the consumption of *G. pentaphyllum* tea (6 g/day for 12 weeks) produced greater reductions in fasting plasma glucose and glycated haemoglobin A1c (HbA1c) and improved insulin-resistance indices compared with placebo tea [76,77]. A subsequent study showed that the addition of *G. pentaphyllum* extract to gliclazide therapy further reduced fasting glucose, post-load glucose, and HbA1c compared with gliclazide plus placebo [76,78]. More recently, Wang et al. [76], in a meta-analysis of eight clinical studies involving 584 patients, reported pooled reductions in fasting plasma glucose, HbA1c, and 2 h postprandial glucose, although heterogeneity among preparations, treatment regimens, and study designs limits the certainty and generalisability of these estimates.

Evidence for effects on body composition is also derived from controlled human studies. Park et al. [79] reported that 12 weeks of supplementation with 450 mg/day of the heat-processed *G. pentaphyllum* extract actiponin reduced the total abdominal fat area, body weight, body fat mass, percentage body fat, and body mass index (BMI) in obese adults compared with placebo. A subsequent 16-week randomised controlled study in overweight adults similarly reported reductions in body weight, BMI, total fat mass, and gynoid fat following supplementation with a standardised *G. pentaphyllum* extract [80]. These effects nevertheless relate to specific standardised preparations and should not be extrapolated automatically to all *Gynostemma* products.

Human data also provide preliminary support for effects on fatigue and exercise metabolism. In a randomised controlled trial involving 100 healthy adults, a gypenoside L-containing *G. pentaphyllum* extract administered for 12 weeks increased VO_2_max and oxygen pulse and reduced selected indices of perceived fatigue [81]. In a smaller randomised crossover study in healthy untrained males, four weeks of *G. pentaphyllum* supplementation improved 20 km cycling time-trial performance and mitochondrial respiration, and it also altered exercise-induced skeletal-muscle AMPK Thr172 phosphorylation [82]. In contrast, proposed benefits in non-alcoholic fatty liver disease (NAFLD) are presently supported mainly by animal and cellular studies showing improvements in hepatic lipid accumulation, insulin resistance, inflammatory signalling, and lipophagy [29,83]. Neuroprotective and anxiolytic effects likewise remain predominantly preclinical [53,84].

### 7.3. Molecular Mechanisms

AMPK represents one of the best-supported metabolic signalling nodes affected by *G. pentaphyllum*, although the molecular route depends on the specific compound or extract investigated. Nguyen et al. [85] demonstrated that the dammarane-type saponins damulin A and damulin B increased AMPK activation in L6 skeletal-muscle cells and concomitantly enhanced glucose uptake, glucose transporter type 4 (GLUT4) translocation, and fatty-acid β-oxidation. These findings provide direct experimental support for AMPK-associated metabolic regulation by chemically defined *Gynostemma* constituents, although they do not establish direct binding to AMPK or a universal requirement for LKB1. The activation of AMPK provides a mechanistically plausible link between *Gynostemma* exposure and the regulation of glucose and lipid metabolism. Downstream AMPK signalling favours energy-generating processes, including glucose uptake and fatty acid oxidation, while restraining energy-consuming anabolic and lipogenic pathways [12,85]. Importantly, evidence for this pathway is not restricted entirely to isolated cells: in healthy men, *G. pentaphyllum* supplementation altered exercise-induced skeletal-muscle AMPK Thr172 phosphorylation and mitochondrial respiration in parallel with improved cycling performance [82]. Nevertheless, these human findings were obtained with a complex leaf extract and therefore cannot be attributed to a single gypenoside. The interpretation of these findings after oral *G. pentaphyllum* administration also requires a consideration of intestinal metabolism. Dammarane-type gypenosides can exhibit low oral bioavailability; for example, gypenoside XLVI showed limited systemic exposure after oral administration in rats [86]. More recent work with *G. pentaphyllum* demonstrated that C-3 deglycosylation by gut microbiota represents a major intestinal metabolic route and can generate metabolites with greater bioavailability and biological potency than the corresponding parent gypenoside [87]. Accordingly, AMPK-related effects observed after oral *G. pentaphyllum* preparations should not be attributed automatically to intact parent gypenosides, and the relative contributions of parent saponins and their intestinal metabolites should be distinguished whenever possible.

Hepatic models indicate additional downstream mechanisms. Gypenoside-rich preparations have been shown to improve lipid accumulation and inflammatory signalling in experimental NAFLD through modulation of the lipopolysaccharide (LPS)/TLR4 pathway [29]. More recently, Zhang et al. [83] demonstrated in high-fat-diet-fed hamsters and lipid-loaded HepG2 cells that gypenosides promoted lipophagy through modulation of the AMPK/mTOR/Unc-51-like autophagy-activating kinase 1 (ULK1) pathway, providing a mechanistic link between energy sensing, autophagy, and hepatic lipid clearance. These findings are consistent with the broader involvement of gypenosides in AMPK, PPAR, PI3K/Akt, and inflammatory signalling described across preclinical models [30,53].

Overall, the available evidence supports AMPK-centred metabolic regulation as a plausible molecular signature of *G. pentaphyllum* but not a single compound–single target mechanism. Differences among isolated gypenosides, saponin-rich fractions, and whole-plant extracts remain an important limitation when translating these pathways to human metabolic outcomes.

## 8. *Centella asiatica* (L.) Urb. (Gotu Kola)

### 8.1. Phytochemical Composition

*Centella asiatica* (L.) Urb., commonly known as Gotu Kola and as Mandukaparni in Ayurvedic medicine, is a creeping perennial herb of the Apiaceae family distributed widely throughout tropical and subtropical regions of Asia, Africa, and other parts of the world. It has a long history of medicinal use, particularly in wound healing and in preparations traditionally employed to support memory and cognitive function [32,33]. Its characteristic phytochemical profile is dominated by ursane-type pentacyclic triterpenes, particularly the triterpenoid glycosides asiaticoside and madecassoside and their corresponding aglycones, asiatic acid and madecassic acid [33,34].

Asiaticoside and madecassoside are among the principal chemical markers of *C. asiatica*, whereas asiatic acid and madecassic acid are formed both naturally in the plant and through the metabolism of their respective glycosides. The relative abundance of these centellosides varies considerably with geographical origin, genotype, plant organ, developmental stage, cultivation conditions, and extraction procedure, which complicates direct comparison among commercial and experimental preparations [34,88]. In addition to triterpenes, *C. asiatica* contains flavonoids, caffeoylquinic acids and other phenylpropanoids, phytosterols, volatile terpenoids, and additional minor constituents that may contribute to its antioxidant and neuroactive properties [33,88].

Standardised preparations have therefore become particularly important for mechanistic and translational research. ECa 233 is a chemically defined *C. asiatica* extract containing a high proportion of triterpenoid glycosides, predominantly madecassoside and asiaticoside, with a specified madecassoside:asiaticoside ratio of approximately 1.5 (±0.5):1 [89,90]. Human pharmacokinetic studies indicate that the oral administration of ECa 233 results in relatively low systemic exposure to the parent glycosides but greater exposure to their respective aglycone metabolites, madecassic acid and asiatic acid, emphasising the importance of metabolism when linking extract composition to biological activity [89]. These data provide a more rigorous basis for evaluating *C. asiatica* than studies employing chemically undefined extracts. Recent experimental evidence further indicates that gut microbiota can contribute directly to this biotransformation. In mice, asiaticoside was hydrolysed by intestinal microbiota to asiatic acid, whereas microbiota depletion markedly reduced metabolite formation and the associated biological response [91]. These findings reinforce the importance of distinguishing exposure to parent glycosides from that to their aglycone metabolites when interpreting the in vivo effects of orally administered *C. asiatica* preparations.

### 8.2. Effects on Human Health

Human evidence for the cognitive and neuropsychological effects of *C. asiatica* remains suggestive but substantially less developed than the preclinical evidence base. In a randomised, double-blind, placebo-controlled study of 28 healthy elderly volunteers, two months of supplementation improved selected measures of working memory, cognitive processing, and mood—particularly at the highest tested dose of 750 mg/day [92]. However, a subsequent systematic review and meta-analysis found no significant overall advantage over placebo across cognitive domains, despite some short-term improvements in mood-related outcomes, highlighting the limited size and consistency of the clinical evidence [93]. Human pharmacokinetic studies of standardised preparations nevertheless provide an important translational foundation by confirming systemic exposure to triterpene metabolites and generally good short-term tolerability [89].

Preclinical evidence is considerably stronger and converges on neurotrophic, synaptic, mitochondrial, and antioxidant mechanisms. In chronic-stress models, *C. asiatica* increased hippocampal BDNF together with TrkB and ERK1/2 signalling, while a phytosomal preparation increased prefrontal BDNF expression and improved recognition memory in rats [14,94]. The standardised extract ECa 233 enhanced hippocampal long-term potentiation ex vivo and, in 3xTg-AD mice, improved fear memory performance together with BDNF/TrkB/ERK-associated synaptic signalling [95,96]. Complementary studies in 5xFAD and aged mice further demonstrated improvements in memory and executive function associated with mitochondrial regulation, synaptic markers, and NRF2-dependent antioxidant responses [88,97].

Preliminary human observations also suggest possible effects on neuroplasticity-related biomarkers: Setiawan et al. [98] reported increased plasma BDNF and changes in cognitive and physical-performance measures in menopausal women receiving *C. asiatica* together with exercise. Because the intervention combined supplementation with physical training, however, the botanical contribution cannot be isolated. Overall, the evidence supports a biologically coherent neuroplastic and neuroprotective potential for *C. asiatica*, but the strongest mechanistic support remains preclinical and has not yet translated into reproducible clinical cognitive efficacy.

### 8.3. Molecular Mechanisms

Neurotrophin-associated signalling is one of the most consistently supported mechanisms underlying the neuroplastic effects of *C. asiatica*, although evidence relates predominantly to complex extracts rather than isolated triterpenes. Across stress and neurodegeneration models, *C. asiatica* preparations increased BDNF expression and TrkB/ERK1/2 signalling, while the standardised extract ECa 233 enhanced hippocampal long-term potentiation and BDNF/TrkB/ERK-associated responses [14,94,95,96]. These findings are compatible with the broader TrkB-dependent ERK and PI3K/Akt signalling involved in neuronal survival and synaptic plasticity. However, the direct activation of a defined protein kinase A (PKA)/calcium/calmodulin-dependent protein kinase (CaMK)–CREB–BDNF pathway by asiaticoside itself has not been established. BDNF/TrkB signalling should therefore be regarded as a downstream molecular signature of neuroactive *C. asiatica* preparations rather than a confirmed single-compound mechanism. Moreover, given the limited systemic exposure to intact asiaticoside and madecassoside after oral administration, the attribution of these intracellular responses specifically to the parent glycosides requires pharmacokinetic evidence distinguishing them from the corresponding aglycones, asiatic acid and madecassic acid [89].

A second mechanistic axis involves NRF2-dependent antioxidant defence, mitochondrial regulation, and neuroinflammation. In aged and β-amyloid-overexpressing mice, aqueous *C. asiatica* extracts enhanced NRF2-regulated antioxidant responses while improving cognitive, mitochondrial, and synaptic outcomes; importantly, the loss of NRF2 abolished the cognitive-enhancing effect of prolonged treatment, supporting a functional role for this pathway [88,97]. Complementary evidence from LPS-stimulated BV2 microglia showed that a standardised extract reduced nitric oxide, TNF-α, IL-6, reactive oxygen species, and lipid peroxidation while increasing Nrf2 and HO-1 expression [99]. These findings are consistent with the broader antioxidant and anti-inflammatory properties reported for asiaticoside and madecassoside [33,34].

Taken together, the mechanistic evidence supports the convergence of BDNF/TrkB-associated neuroplasticity and NRF2-dependent redox regulation with ERK/PI3K-Akt, mitochondrial, and neuroimmune pathways acting as interconnected downstream components. This provides a coherent biological framework for the neuroprotective effects observed experimentally, but causal confirmation in humans remains insufficient.

## 9. *Glycyrrhiza glabra* L. (Liquorice)

### 9.1. Phytochemical Composition

*Glycyrrhiza glabra* L. (Fabaceae), commonly known as liquorice or licorice, has been used medicinally for millennia in European, Middle Eastern, and Asian traditions. The principal medicinal material consists of the dried roots and stolons, although related species, particularly *G. uralensis* Fisch. and *G.* inflata Batalin, are also used in traditional and pharmacopoeial preparations. Their phytochemical profiles differ substantially with species, geographical origin, cultivation, and processing, making botanical authentication and chemical standardisation essential for pharmacological interpretation [17,36,100].

The characteristic triterpenoid saponin is glycyrrhizin (glycyrrhizic acid), which is composed of 18β-glycyrrhetinic acid linked to two glucuronic-acid residues. Its concentration varies widely among raw materials and preparations. Following oral ingestion, glycyrrhizin is extensively hydrolysed by the intestinal microbiota to 18β-glycyrrhetinic acid and subsequently metabolised further with these metabolites contributing substantially to both systemic pharmacological activity and toxicity [17,100]. The phenolic fraction is similarly diverse and includes liquiritin, liquiritigenin, isoliquiritin, isoliquiritigenin, glabridin, glabrene, and other flavonoid and prenylated phenolic constituents [36,100]. Glabridin and glabrene exhibit oestrogen-like activity in experimental systems, although this does not establish selective ERβ agonism or clinical oestrogenic efficacy in humans [101].

Of particular mechanistic relevance, 18β-glycyrrhetinic acid and related glycyrrhizin metabolites inhibit 11β-hydroxysteroid dehydrogenase type 2 (11β-HSD2), reducing the conversion of active cortisol to cortisone in mineralocorticoid-sensitive tissues. This interaction provides a direct molecular link between liquorice exposure and peripheral glucocorticoid metabolism while simultaneously explaining the mineralocorticoid-like adverse effects associated with excessive or prolonged intake [16,17].

### 9.2. Effects on Human Health

Unlike several other botanicals discussed in this review, the proposed relevance of *G. glabra* to stress physiology derives primarily from its well-established effects on peripheral glucocorticoid metabolism rather than from clinical evidence of improved stress resilience. Liquorice-derived glycyrrhizin and glycyrrhetinic acid inhibit cortisol-to-cortisone conversion and thereby increase local glucocorticoid availability in 11β-HSD2-expressing tissues [17]. This pharmacodynamic effect is robust, but it should not itself be interpreted as evidence of an adaptogenic health benefit.

Clinical evidence for other applications is heterogeneous and strongly dependent on the formulation, dose, and route of administration. Glycyrrhizin- and deglycyrrhizinated-liquorice preparations have been investigated in gastrointestinal, hepatic, oral, and dermatological disorders, but the overall quality and consistency of evidence remain limited [17,36]. Historical studies of deglycyrrhizinated liquorice in peptic ulcer disease were inconsistent, and a recent meta-analysis found no significant pooled benefit despite trends towards improved healing and pain [102]. Parenteral glycyrrhizin has also reduced serum aminotransferase activities in some studies of chronic viral hepatitis, although evidence for direct antiviral efficacy is weaker and cannot be extrapolated to oral liquorice preparations [17,36]. Neuroprotective effects remain preclinical: in cerebral-ischaemia models, glycyrrhizin reduced inflammatory injury, inhibited glycyrrhizin reduced inflammatory injury, and inhibited high-mobility group box 1 (HMGB1) phosphorylation and release [103].

Critically, the same glucocorticoid-modulating mechanism that underpins pharmacological interest also defines the principal safety limitation. Chronic glycyrrhizin exposure can enhance cortisol-dependent mineralocorticoid-receptor activation, suppress renin and aldosterone, promote sodium retention and potassium loss, and increase blood pressure [17,104]. These effects are clinically relevant even at moderate exposure: 100 mg/day glycyrrhizic acid for two weeks increased home systolic blood pressure and markedly suppressed renin and aldosterone in healthy adults [105], while a meta-analysis of eight randomised trials involving 541 participants confirmed significant increases in both systolic and diastolic blood pressure [106]. Thus, increased glucocorticoid availability should be regarded as a dose-dependent pharmacological effect with a narrow boundary between potential therapeutic modulation and clinically relevant toxicity.

### 9.3. Molecular Mechanisms

The best-established molecular mechanism linking *G. glabra* with glucocorticoid regulation is the inhibition of 11β-hydroxysteroid dehydrogenase type 2 (11β-HSD2). This NAD^+^-dependent enzyme protects mineralocorticoid receptors in tissues such as the distal nephron by converting active cortisol to cortisone. Following oral liquorice intake, glycyrrhizin is metabolised by intestinal microbiota to 18β-glycyrrhetinic acid and related metabolites that inhibit 11β-HSD2, thereby increasing the local ratio of cortisol to cortisone and permitting cortisol-dependent mineralocorticoid-receptor activation [16,17]. The resulting sodium and water retention, potassium loss, and suppression of renin and aldosterone provide a coherent mechanistic explanation for liquorice-induced pseudohyperaldosteronism [17,104,105]. Importantly, this mechanism reflects peripheral glucocorticoid regulation rather than enhancement of the HPA axis function and should not be interpreted as evidence of improved stress adaptation. Its relevance to the adaptogen concept is therefore pharmacologically distinctive but intrinsically ambivalent: the same pathway that alters endogenous glucocorticoid exposure also underlies dose-dependent hypertension, hypokalaemia, and fluid retention. This duality makes *G. glabra* an important example of why mechanistic activity and adaptogenic benefit cannot be treated as equivalent.

A second, mechanistically distinct pathway involves HMGB1. Glycyrrhizin binds directly to HMGB1 and inhibits its extracellular inflammatory activities [107], while in post-ischaemic rat brain, it reduced HMGB1 phosphorylation and release, microglial activation, inflammatory signalling, and neuronal injury [103]. Other liquorice constituents, including liquiritigenin, isoliquiritigenin, glabridin, and glabrene, display additional antioxidant, anti-inflammatory, and oestrogen-like activities in experimental systems [36,101]. However, these effects remain predominantly preclinical and are secondary to the substantially stronger translational evidence supporting the 11β-HSD2–glucocorticoid pathway.

## 10. Critical Perspective: Challenges and Future Directions

The molecular mechanisms discussed throughout this review provide a strong biological rationale for several proposed next-generation adaptogens, but mechanistic plausibility should not be equated with demonstrated clinical efficacy. Across the seven species, the evidence base remains uneven: detailed molecular pathways are frequently derived from cellular and animal models, whereas human studies are fewer, often relatively small, and commonly use preparations that differ in composition, dose, and degree of phytochemical standardisation. The principal human intervention studies underlying this translational assessment are summarised in Table 1, which highlights the heterogeneity in preparation, study design, endpoints, safety reporting, and evidential limitations across the seven proposed next-generation adaptogens.

The gap between molecular evidence and human target engagement is particularly apparent for several compounds reviewed here. Macamides directly inhibit fatty acid amide hydrolase in biochemical and cellular systems, supporting FAAH as a plausible molecular target of maca [10,19]. However, human studies have not yet demonstrated the complete sequence from macamide exposure through FAAH inhibition and altered endocannabinoid signalling to a clinically relevant outcome. Similarly, *Centella asiatica* produced selected improvements in cognition and mood in a small trial in elderly volunteers [92], whereas much of the mechanistic evidence linking the plant to BDNF/TrkB signalling derives from experimental models [14,94]. In contrast, the study of *Gynostemma pentaphyllum* by Nayyar et al. [82] illustrates a more informative translational design by combining a human functional endpoint with measurements of skeletal-muscle mitochondrial respiration and AMPK signalling.

This distinction is equally important when evaluating biomarkers. Changes in telomerase activity or telomere length cannot by themselves establish an anti-ageing benefit; increased circulating BDNF does not directly demonstrate hippocampal neuroplasticity; and AMPK activation does not necessarily predict clinically meaningful metabolic improvement. Mechanistic biomarkers are most informative when they form part of a coherent causal sequence linking a chemically characterised preparation with systemic exposure, molecular target engagement, a predicted biological response, and a clinically relevant endpoint.

Safety must be integrated into the same framework. *Glycyrrhiza glabra* provides a particularly clear example because the inhibition of 11β-HSD2 is pharmacologically well established, yet the resulting increase in local cortisol activity may also promote mineralocorticoid-receptor activation, sodium retention, potassium loss, and increased blood pressure. Thus, a well-defined molecular mechanism is not necessarily beneficial and may instead reveal a narrow therapeutic window. Similar attention should be given to dose, duration, cumulative exposure, herb–drug interactions, and vulnerable populations for all proposed adaptogens.

Future clinical studies should therefore prioritise adequately powered and prospectively registered trials using standardised and chemically characterised preparations, predefined primary outcomes, appropriate comparators, sufficient intervention duration, and transparent adverse-event reporting. Reporting should additionally follow established guidance for herbal interventions, including rigorous botanical authentication and quantitative phytochemical characterisation of the investigated preparations [109,110]. Mechanism-aligned biomarkers should be selected according to the proposed mode of action—for example, FAAH activity and endocannabinoid mediators for maca, telomerase-related measures for cycloastragenol-containing preparations, BDNF-associated endpoints for *C. asiatica*, and AMPK-related metabolic markers for cordycepin- or gypenoside-containing interventions.

Ultimately, progress in adaptogen research will depend less on accumulating additional positive observations than on demonstrating a reproducible relationship between a chemically defined preparation, its systemic exposure, engagement of the proposed molecular target, the resulting biological response, and a clinically meaningful outcome.

### 10.1. Standardisation and Bioavailability Challenges

Chemical standardisation remains one of the major practical challenges in adaptogen research. The concentrations of key bioactive or marker compounds discussed in this review—including macamides, astragaloside IV, cordycepin, 20-hydroxyecdysone, gypenosides, asiaticoside, and glycyrrhizin—may vary substantially with species or chemotype, geographical origin, cultivation conditions, plant or fungal material, harvest stage, post-harvest processing, and extraction methodology. This variability may result in preparations with markedly different pharmacological properties despite being marketed under the same botanical or fungal name. In *Cordyceps*, for example, cordycepin content differs considerably between *Ophiocordyceps sinensis* and *C. militaris* and is additionally influenced by cultivation and extraction conditions [60]. Reported concentrations in *C. militaris* preparations span several-fold ranges rather than supporting a single representative value. Cordycepin is subject to rapid ADA-mediated deamination to 3′-deoxyinosine, which further limits systemic exposure to the parent nucleoside after administration. The experimental inhibition of ADA can prolong cordycepin exposure, but pharmacokinetic findings obtained under such conditions should not be extrapolated directly to conventional oral *C. militaris* preparations [66,67].

Bioavailability introduces an additional source of variability. Following oral administration, glycyrrhizin is extensively hydrolysed by intestinal microbiota to 18β-glycyrrhetinic acid, which is a substantially more potent inhibitor of 11β-HSD2; gastrointestinal and enterohepatic processes therefore influence systemic exposure to the active metabolite [17]. Astragaloside IV also exhibits very low oral bioavailability, which is primarily because of poor intestinal permeability rather than established extensive first-pass glucuronidation [111]. Oral exposure to dammarane-type gypenosides and *C. asiatica* triterpenoid glycosides is likewise strongly influenced by intestinal biotransformation. Gypenoside XLVI exhibits low oral bioavailability, while the gut microbiota-mediated deglycosylation of *G. pentaphyllum* saponins can generate metabolites with greater bioavailability and biological potency than their parent compounds [86,87]. Human pharmacokinetic studies of ECa 233 similarly demonstrate low systemic exposure to madecassoside and asiaticoside but substantially greater exposure to their corresponding aglycones, madecassic acid and asiatic acid [89]. Interindividual differences in gut microbial metabolism may therefore contribute to variability in systemic exposure and biological response. Substantial person-to-person variation in the microbial transformation of structurally related dammarane saponins has been demonstrated using human gut bacterial samples [112]. However, the extent to which such microbiota-dependent variability accounts for differences in clinical response to *G. pentaphyllum* or *C. asiatica* remains to be established.

Human pharmacokinetic data for macamides remain limited. Formulation approaches such as phytosomes, self-emulsifying systems, and nanoparticle-based delivery may improve exposure, but their clinical value requires direct confirmation. For *C. asiatica*, phytosomal delivery has shown biological activity in experimental models, but the available Sbrini et al. [14] study was conducted in rats and therefore does not constitute clinical validation.

### 10.2. Safety, Herb–Drug Interactions, and Regulatory Considerations

The designation of adaptogens as generally safe reflects their historical use and favourable safety profiles at commonly studied doses, but it does not exempt them from rigorous safety assessment in specific populations or at high exposures.

For *G. glabra*, excessive glycyrrhizin exposure is associated with pseudoaldosteronism, including sodium retention, hypokalaemia and hypertension, and particular caution is warranted with medicines that may aggravate electrolyte imbalance [17,113]. Glycyrrhizin and glycyrrhetinic acid may also modulate drug-metabolising enzymes and transport proteins, including CYP3A and P-glycoprotein, indicating potential for clinically relevant herb–drug interactions [114].

For *A. membranaceus*, its immunomodulatory polysaccharides warrant caution in autoimmune conditions, although direct clinical evidence demonstrating an exacerbation of autoimmune disease remains limited [115]. Telomerase activation by cycloastragenol-related preparations has also raised theoretical concerns regarding oncological safety. Current human studies of TA-65 are too limited in size and duration to establish long-term cancer risk, and continued surveillance is therefore warranted [23].

For *R. carthamoides*, experimental evidence indicates that 20-hydroxyecdysone does not act through the classical androgen-receptor pathway, although human safety data remain limited [15,28]. Since 2020, 20-hydroxyecdysone has been included in the World Anti-Doping Agency (WADA) Monitoring Programme and remains monitored in 2026, but it is not currently included in the Prohibited List [116].

Comparable caution is warranted for the remaining candidates. Maca preparations have generally been well tolerated in short-term human studies; for example, red and black *L. meyenii* extracts administered at 3 g/day for 12 weeks produced no serious adverse effects [117]. However, these findings do not establish the safety of prolonged exposure or use in populations not represented in the available trials. Human studies of *C. militaris* are similarly limited in duration, ranging from the single-dose administration of a standardised extract to short supplementation periods, and therefore do not define a long-term safety threshold or a clinically established herb–drug interaction profile [62,63]. For *G. pentaphyllum*, short controlled studies have reported no major adverse events, including the administration of 450 mg/day for 4 weeks [82], while a systematic review of randomised trials in dyslipidaemia found fewer adverse events than with conventional lipid-lowering agents and suggested that use beyond 8 weeks appeared generally safe; nevertheless, the certainty of this evidence was low [118]. Standardised *C. asiatica* extract ECa 233 was well tolerated after single and repeated oral administration at 250 and 500 mg in healthy volunteers [89], although rare cases of clinically apparent hepatotoxicity have been reported after oral *C. asiatica* use [119]. Particular caution is therefore warranted in individuals with pre-existing liver disease. Across these candidates, evidence regarding prolonged use, pregnancy and lactation, other vulnerable populations, and systematic herb–drug interaction assessment remains insufficient; the absence of reported toxicity should therefore not be interpreted as established long-term safety.

Drug interactions involving CYP enzymes, transport proteins, or hormonal signalling remain plausible for several of the phytochemicals reviewed, but systematic interaction studies are still limited. Regulatory frameworks in the European Union, United States, and China differ substantially in the classification and oversight of botanical supplements, contributing to variability in product standardisation and the evidence required to support health-related claims.

### 10.3. Future Research Priorities

Future research on next-generation adaptogens should prioritise studies that connect clinical outcomes with direct evidence of molecular target engagement. Mechanism-aligned biomarkers should therefore be incorporated into appropriately designed trials but selected according to the strength of the underlying evidence rather than theoretical plausibility alone. For example, FAAH activity and endocannabinoid-related mediators are relevant to the macamide hypothesis for *L. meyenii* [10,19], whereas skeletal-muscle AMPK signalling has already been evaluated in humans receiving *G. pentaphyllum* [82]. Comparable approaches could be developed for the other adaptogens provided that biomarker selection reflects the experimentally supported mechanism and is interpreted alongside clinically meaningful endpoints.

Pharmacokinetic characterisation is equally important. For several principal bioactives, information on systemic exposure, metabolite formation, and interindividual variability remains incomplete, limiting the interpretation of dose–response relationships. Future studies should therefore integrate pharmacokinetic and pharmacodynamic measurements and distinguish effects of parent compounds from those of biologically active metabolites. Microbiome-dependent metabolism may be particularly relevant for compounds such as glycyrrhizin, but microbiome analysis should be incorporated when mechanistically justified rather than applied routinely to all interventions.

Finally, future trials should use chemically standardised preparations, adequate statistical power, clinically relevant intervention periods, and rigorous safety monitoring. Combination products should be evaluated experimentally rather than assumed to be synergistic on the basis of overlapping molecular pathways. The principal objective should be to establish a reproducible relationship between defined exposure, molecular target engagement, measurable biological response, and clinically meaningful benefit.

## 11. Conclusions

This review positions seven selected species—six botanicals and one fungus—as candidates for a pathway-oriented extension of adaptogen pharmacology. Collectively, they illustrate molecular mechanisms involving endocannabinoid regulation, telomere and senescence biology, AMPK-dependent metabolic control, non-androgenic anabolic signalling, neurotrophin and antioxidant pathways, immune modulation, and peripheral glucocorticoid metabolism. Several of these pathways, particularly those related to telomere maintenance and cellular senescence, mitochondrial function, metabolic regulation, neuroplasticity, and glucocorticoid homeostasis, also intersect with biological processes relevant to healthy ageing and the maintenance of functional resilience. However, their contribution to extended healthspan or healthy longevity remains to be demonstrated in humans.

The principal challenge is therefore no longer the identification of additional molecular targets but rather their translation into reproducible human effects. Mechanistic plausibility alone does not establish adaptogenic efficacy, and improvements in experimental biomarkers cannot be assumed to predict clinically meaningful benefit. Progress will require chemically characterised preparations, adequate pharmacokinetic and bioavailability data, direct evidence of target engagement, mechanism-aligned biomarkers, clinically relevant endpoints, and safety assessment integrated within the same experimental framework.

Accordingly, the value of the next-generation adaptogen concept lies not in expanding the list of substances labelled as adaptogens but rather in raising the evidential standard by which such candidates are evaluated. Only when molecular mechanism, systemic exposure, biological response, clinical benefit, and safety can be linked coherently should a candidate be considered a substantiated extension of adaptogenic pharmacology rather than a mechanistically promising but incompletely translated natural product.

## Figures and Tables

**Figure 1:**
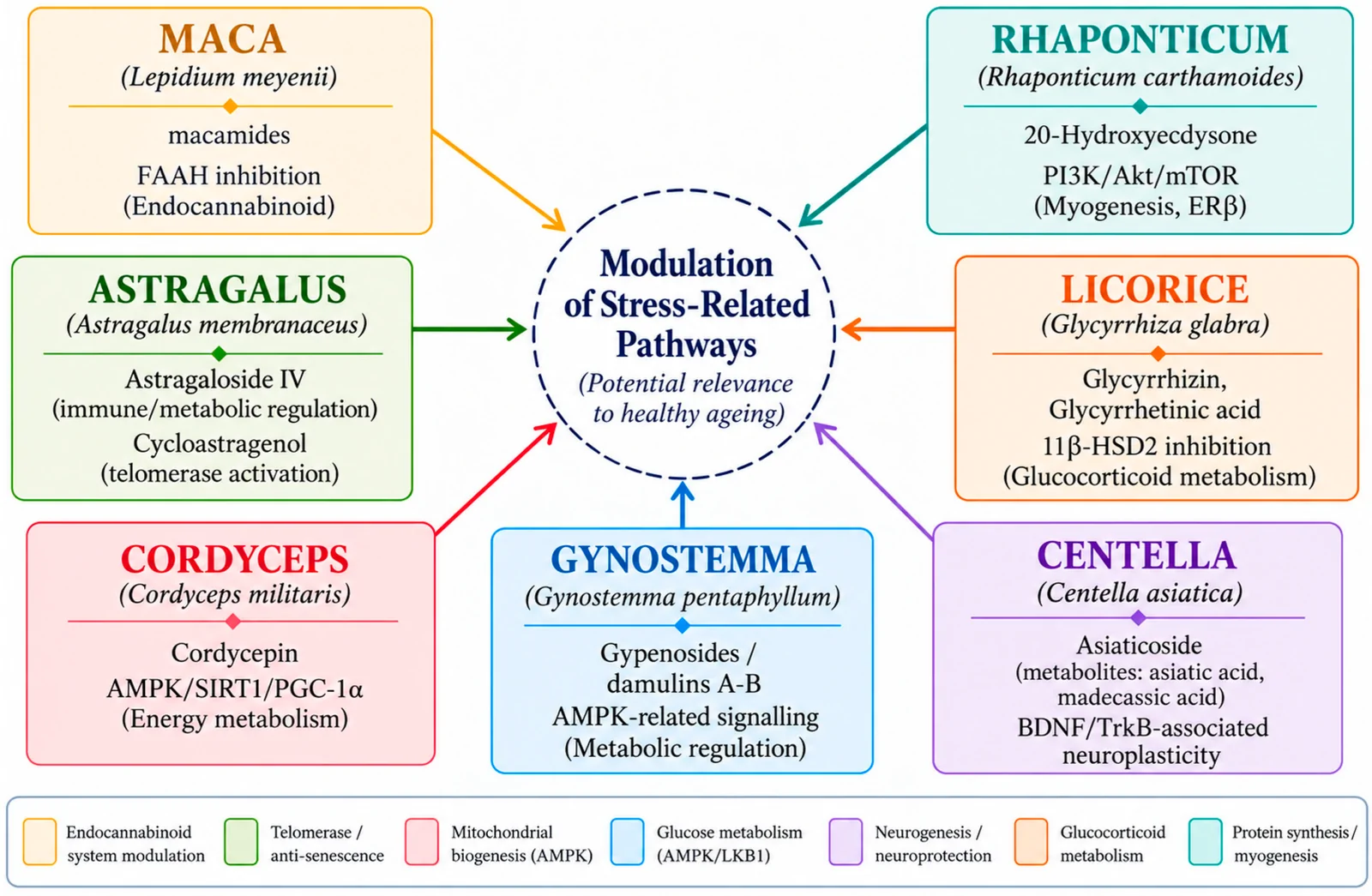
Overview of seven proposed next-generation adaptogens, their principal bioactive compounds, and key molecular pathways through which they may support stress resilience and homeostatic adaptation with potential relevance to healthy ageing.

**Figure 2:**
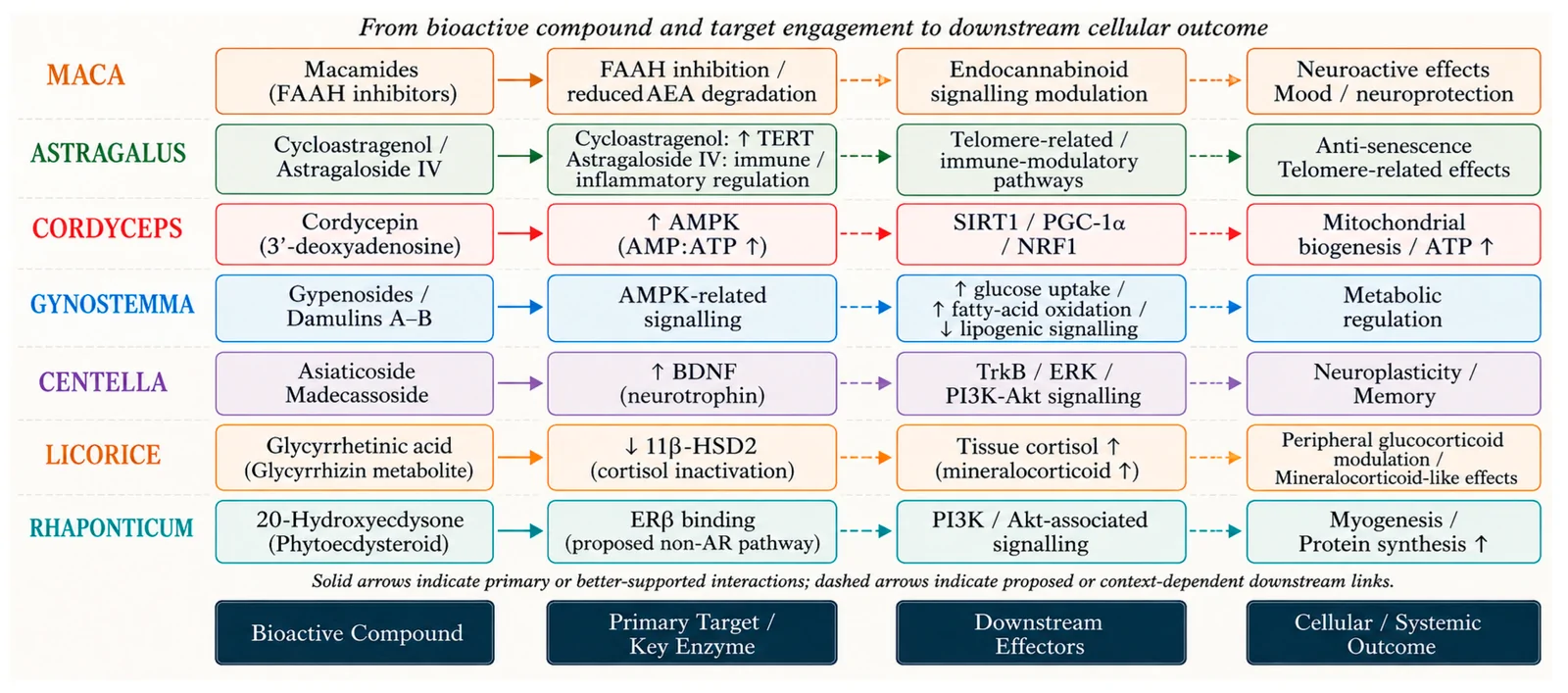
Proposed molecular pathways associated with seven next-generation adaptogens, linking representative bioactive compounds with primary molecular targets, downstream effectors, and cellular or systemic outcomes. Solid arrows indicate primary or better-supported interactions, whereas dashed arrows indicate proposed, indirect, or context-dependent downstream relationships. Upward and downward arrows (↑ and ↓) indicate an increase and decrease, respectively.

**Table 1:** Summary of key human intervention studies evaluating the seven proposed next-generation adaptogens.

Species/Study	Preparation/Standardisation	Study Design and Exposure	Main Endpoint and Result	Safety	Key Limitation
*L. meyenii* Gonzales et al. [108]	Gelatinised maca; no macamide standardisation	1.5 or 3 g/day, 12 wk; *n* = 57; DB-RCT, placebo	Sexual desire ↑; no hormonal changes	Limited AE reporting	Subjective outcome; no mechanistic confirmation
*Astragalus*-derived TA-65 Salvador et al. [55]	Proprietary TA-65	250 or 1000 U (8 or 32 mg active ingredient); 1 yr; *n* = 117; DB-RCT, placebo	Median telomere length ↑ only at low dose	No major safety signal	Biomarker endpoint; no clear dose response
*C. militaris* Farzan & Koushkie Jahromi [63]	Standardised extract; ≥15% β-glucans, ≥0.25% cordycepin	1 g, single dose; *n* = 12; crossover RCT, placebo	Resting VO_2_ ↑; reaction time improved; peak VO_2_ unchanged	Acute exposure only	Very small male sample; single dose
20-Hydroxyecdysone Isenmann et al. [69]	Commercial 20E preparation; measured content lower than label	~12 or 48 mg 20E/day; 10 wk; *n* = 46; training trial	Muscle mass and bench-press performance ↑	No liver/kidney signal	Small groups; product not *R. carthamoides*
20-Hydroxyecdysone Wilborn et al. [70]	Commercial phytoecdysteroid preparation	200 mg/day; 8 wk; *n* = 45; DB-RCT, placebo	No effect on body composition, strength or hormones	Limited reporting	Preparation/dose inconsistencies
*G. pentaphyllum * Huyen et al. [77]	Botanical tea; no individual gypenoside standardisation	6 g/day, 12 wk; *n* = 24; RCT, placebo tea	Fasting glucose, HbA1c and insulin resistance improved	No major AEs reported	Very small T2D sample
*G. pentaphyllum * Nayyar et al. [82]	Complex leaf extract	450 mg/day, 4 wk; *n* = 16; crossover RCT	Exercise performance and mitochondrial respiration improved; AMPK altered	No major signal highlighted	Very small young-male sample
*C. asiatica* Wattanathorn et al. [92]	Standardised extract containing asiaticoside/asiatic acid	250–750 mg/day, 2 mo; *n* = 28; DB-RCT, placebo	Selected cognition/mood outcomes improved	AE reporting limited	Very small dose groups; multiple outcomes
*G. glabra * af Geijerstam et al. [105]	Product standardised for glycyrrhizic acid	100 mg/day, 2 wk; *n* = 28; crossover control	Systolic BP ↑; renin/aldosterone ↓	BP/RAAS effect is safety signal	Short-term healthy-volunteer study

Abbreviations and units: 20E, 20-hydroxyecdysone; AEs, adverse events; AMPK, AMP-activated protein kinase; BP, blood pressure; DB-RCT, double-blind randomised controlled trial; HbA1c, glycated haemoglobin A1c; *n*, number of participants; RAAS, renin–angiotensin–aldosterone system; RCT, randomised controlled trial; T2D, type 2 diabetes; VO_2_, oxygen uptake; g/day, grams per day; mg/day, milligrams per day; wk, week(s); mo, month(s); yr, year(s); U, units of the TA-65 formulation; ↑, increase or improvement; ↓, decrease.

## Data Availability

No new data were created or analysed in this study. Data sharing is not applicable to this article, as this is a review based on previously published literature.
